# Metabolic Profiling of Plasma from Benign and Malignant Pulmonary Nodules Patients Using Mass Spectrometry-Based Metabolomics

**DOI:** 10.3390/metabo3030539

**Published:** 2013-07-04

**Authors:** Liang Gao, Zongmei Wen, Chunyan Wu, Tao Wen, Choon Nam Ong

**Affiliations:** 1 NUS Environmental Research Institute, National University of Singapore, 5A Engineering Drive 1, Singapore 117411, Singapore; E-Mail: erigl@nus.edu.sg; 2 Department of Anesthesiology, Shanghai Pulmonary Hospital, Tongji University School of Medicine, Shanghai 200433, China; E-Mails: wzm1103@126.com (Z.W.); wuchunyan581@sina.com (C.W.); 3 Saw Swee Hock School of Public Health, National University of Singapore, 16 Medical Drive, Singapore 117597, Singapore; E-Mails: wentao5281@163.com (T.W.); ephocn@nus.edu.sg (C.N.O.)

**Keywords:** metabolomics, solitary pulmonary nodules, plasma, biomarkers, mass spectrometry

## Abstract

Solitary pulmonary nodule (SPN or coin lesion) is a mass in the lung and can be commonly found in chest X-rays or computerized tomography (CT) scans. However, despite the advancement of imaging technologies, it is still difficult to distinguish malignant cancer from benign SPNs. Here we investigated the metabolic profiling of patients with benign and malignant pulmonary nodules. A combination of gas chromatography/mass spectrometry (GC/MS) and liquid chromatography/mass spectrometry (LC/MS) was used to profile the plasma metabolites in 17 patients with malignant SPNs, 15 patients with benign SPNs and 20 healthy controls. The metabolic profiles were assayed using OPLS-DA, and further analyzed to identify marker metabolites related to diseases. Both GC/MS- and LC/MS-derived models showed clear discriminations in metabolic profiles among three groups. It was found that 63 metabolites (12 from GC/MS, 51 from LC/MS) contributed to the differences. Of these, 48 metabolites showed same change trend in both malignant and benign SPNs as compared with healthy controls, indicating some common pathways including inflammation and oxidative injury shared by two diseases. In contrast, 14 metabolites constituted distinct profiles that differentiated malignant from benign SPNs, which might be a unique biochemical feature associated with lung cancer. Overall, our data suggested that integration of two highly sensitive and complementary metabolomics platforms could enable a comprehensive metabolic profiling and assist in discrimination malignant from benign SPNs.

## 1. Introduction

Solitary pulmonary nodules (SPNs), defined as relatively spherical opacities seen in the lung that measure up to 3 cm or less in diameter, are quite common in clinical pulmonary screening practice [1]. Most SPNs are detected incidentally on chests X-rays or computerized tomography (CT) scans that are usually performed for some other purpose [2]. It is reported that the prevalence of SPNs is so high that around half of smokers over the age of 50 would have the nodules. Of much note, SPNs can be either benign (non-cancerous) or malignant (lung cancer). The early differentiation of malignant from benign SPNs is extremely critical because it represents a totally different approach regarding treatment and prediction of prognosis [1,3]. In most cases, malignant nodule is a potentially curable form of lung cancer that is usually at early clinical stage and less aggressive biologically.

For benign SPNs, the most common causes include chronic smoking, infections such as histoplasmosis or tuberculosis, a small collection of normal cells (hamartoma), intrapulmonary lymph node, and vascular abnormalities [4]. Benign SPNs usually require no treatment. In contrast, malignant SPNs generally represent an early stage of lung cancer or metastatic cancers that have spread to the lung from other regions of the body [5,6]. As is known, lung cancer is the leading cause of cancer death around the world, accounting for more deaths annually than liver, colon, breast, and prostate cancers [7,8]. The survival rates of lung cancer remain frustratingly low at 15% at 5 years, but an early diagnosis and treatment of lung cancer may lead to 5-year survival rates up to 70%–80% [7]. Therefore, it is of great clinical significance to engender an early and accurate diagnosis as to whether the pulmonary nodules are malignant or benign when they are identified.

Nevertheless, to date, it is still most challenging to distinguish malignant from benign SPNs, as most patients are usually asymptomatic. Even if symptoms are present, they may just include cough or coughing up blood and being short of breath, which are inherently non-specific and do not draw considerable attention [6]. Indeed, a substantial percentage of patients undergo surgery that ultimately reveals only benign nodules. So far, chest X-ray is usually performed to identify pulmonary nodules as a routine screening test. CT scan and positron emission tomography (PET) scan are both more sensitive and specific than chest X-ray, and are thus employed to do further imaging when pulmonary nodules are seen on chest X-rays [2,9]. However, CT and PET scans are so sensitive that sometimes they identify too many small benign nodules. It is therefore not recommended to apply CT or PET scans in every case [1,8]. In fact, clinical and imaging characteristics cannot reliably distinguish malignant from benign SPNs in most patients. Only biopsy of the nodules via bronchoscopy or lung surgery is the “gold standard” diagnostic test and can confirm whether the nodules are cancerous or not [2,3]. However, owing to its invasive nature and the high costs involved, most patients are reluctant to undergo tissue biopsy. Overall, there is an urgent need for efficient strategies with high diagnostic accuracy that are able to differentiate malignant from benign SPNs in a minimally invasive manner.

Metabolomics is defined as the quantitative measurement of the dynamic metabolic responses of living systems to pathophysiological stimuli, genetic modification or environmental factors [10]. Metabolomics focuses mainly on low molecular weight metabolites (e.g., < 1000 amu) such as amino acids, fatty acids, lipids and carbohydrates, which are end products of many biochemical pathways and are closely involved in cellular physiology, structure and signaling [11]. More recently, it has been recognized that the alterations of small metabolites could serve as a new biochemical hallmark related to a variety of diseases including lung cancer, which have shown potential in disease diagnosis, biomarker screening and characterization of the associated biological pathways [10,12,13].

It is hypothesized that there are different plasma metabolic profiles in malignant and benign SPNs, which may be useful for their discrimination. More recently, metabolomic technologies based on nuclear magnetic resonance (NMR), GC/MS, LC/MS have been well documented for application in various research fields [14,15,16,17]. In our previous studies, we have well established an integrated utilization of GC/MS and LC/MS approach to explore metabolic alterations in colorectal cancer and diabetes [18,19]. In the present study, owing to the difficulty of obtaining suitable clinical samples with confirmed diseases, we conducted an exploratory study using GC/MS- and LC/MS-based metabolomics in combination in order to study the profiles of metabolites among 17 patients with malignant and 15 benign SPNs, as well as 20 healthy controls. It is hoped that the identified metabolites can be used for early detection of the malignancy of nodules.

## 2. Results and Discussion

### 2.1. Patient Characteristics

**Figure 1:**
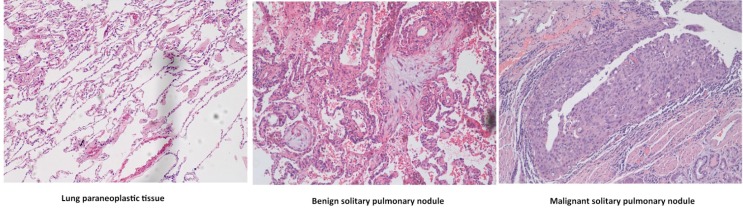
Representative microscopic images of normal lung tissue, benign and malignant solitary pulmonary nodules (SPNs). Scale bars: 100 μM.

Patients with SPNs were confirmed as malignant or benign by histological analysis (Figure 1). All patients and healthy controls were comparable except that patients were older than controls (Table 1), but such a difference did not have significant discriminating power in the classification of groups since we used a linear regression model to exclude the possible interfering factors.

**Table 1:** Characteristics of patients with pulmonary benign nodules, patients with malignant nodules, and healthy controls.

	Healthy controls	Pulmonary benign	Pulmonary malignant
		nodules	nodules
No. of subjects	20	15	17
Gender	Male	Male	Male
Age range (median)	29–50 (38)	33–79 (59)	49–67(60)
Smoking habits	Smokers 6	Smokers 9	Smokers 17
	Non-smokers 14	Non-smokers 6	Non-smokers 0
Nodule size (average)		1.9 cm	2.2 cm
Histological type			Squamous cell carcinoma
Cancer stage			Ⅰ

### 2.1. GC/MS Analysis

**Figure 2:**
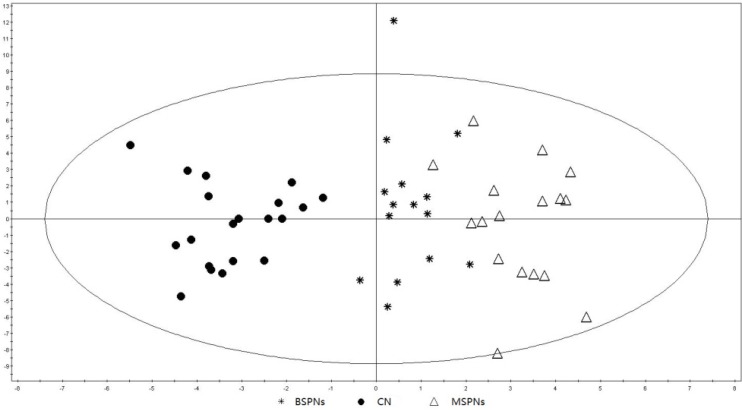
OPLS-DA score plot for malignant SPNs patients (n = 17), benign SPNs patients (n = 15) and healthy controls (CN, n = 20) based on GC/MS data (R^2^Y = 0.887 and Q^2^ = 0.759). The x-axis and y-axis indicate the first principal component and second principal component, respectively.

**Figure 3:**
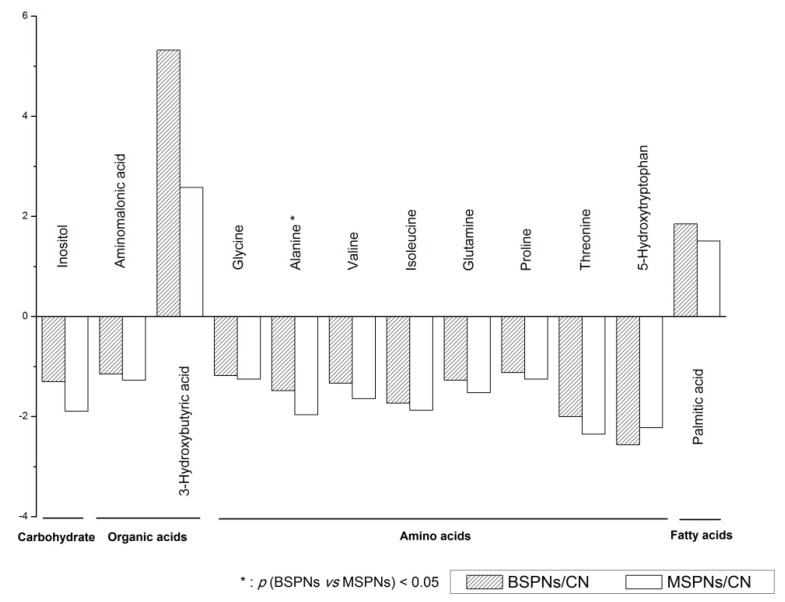
Comparison of metabolomes from healthy controls, malignant and benign SPNs analyzed using GC/MS. These metabolites and small molecules are arbitrarily classified into carbohydrate, organic acids, amino acids and fatty acids.

After peak alignment, the variables derived from GC/MS were used to construct orthogonal projections to latent structures discriminate analysis (OPLS-DA) model. The results clearly indicated that all patients could be clearly segregated from healthy controls, except that there was a slight overlapping between malignant and benign SPNs groups (Figure 2). The performance parameters of R^2^Y = 0.887 and Q^2^ = 0.759 indicated that the model was reliable and highly predictive. We then identified 12 marker metabolites with VIP > 1, which differentiated cases from controls, as shown in Figure 3. Among which, alanine was found to contribute to the differentiation between benign and malignant SPNs patients (*p* < 0.05 and AUC ≥ 0.7, as shown in the Supplementary Table). Other metabolites were observed to have the same trends for both malignant and benign SPNs patients as compared to healthy controls.

*: Significant at *p* < 0.05 between malignant and benign SPNs.

### 2.2. LC/MS Analysis

**Figure 4:**
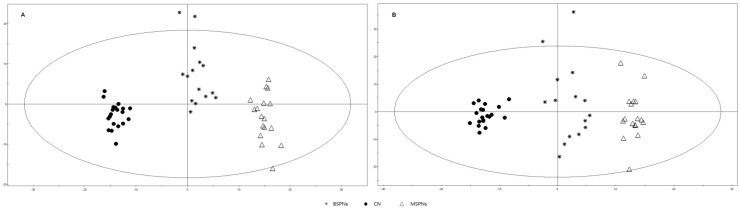
OPLS-DA score plot for malignant SPNs patients (n = 17), benign SPNs patients (n = 15) and healthy controls (CN, n = 20) based on LC/MS data. A: positive mode (R^2^Y = 0.982, Q^2^ = 0.838), B: negative mode (R^2^Y = 0.967 and Q^2^ = 0.815). The x-axis and y-axis indicate the first principal component and second principal component, respectively.

The variables collected in either positive or negative modes of LC/MS were used to construct the OPLS-DA models. The results showed that the plasma profiles for patients with malignant SPNs, patients with benign SPNs and healthy controls, were very well separated in both positive and negative modes (Figure 4). The positive mode (Figure 4A) showed performance statistics of R^2^Y = 0.982 and a good predictive parameter Q^2^ = 0.838, whereas the negative mode (Figure 4B) also showed a very good performance statistics of R^2^Y = 0.967 and Q^2^ = 0.815, suggesting that the models were valid and highly predictive.

**Figure 5:**
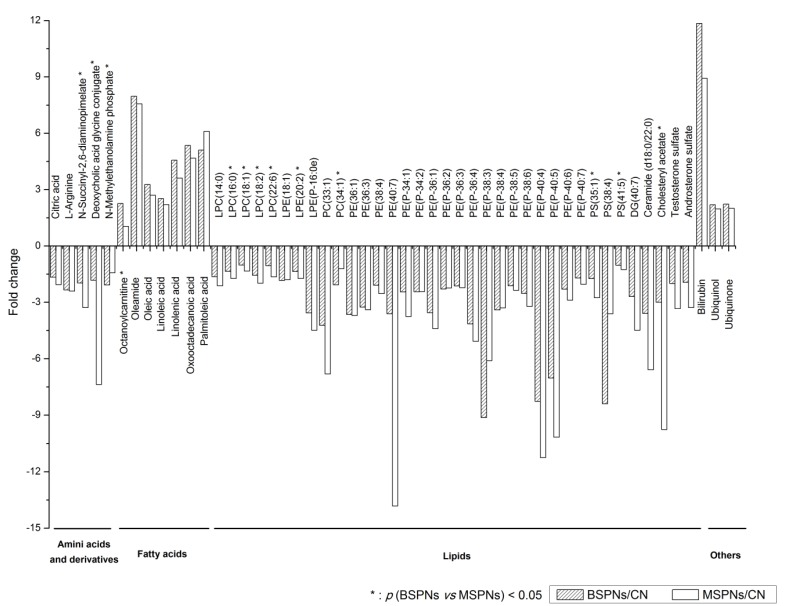
Comparison of metabolomes from healthy controls, malignant and benign SPNs analyzed using LC/MS. These metabolites and small molecules are arbitrarily classified into amino acids and derivatives, fatty acids, lipids and others.

The selected marker metabolites on the robust list were further analyzed by tandem mass or reference standards. A total of 51 metabolites including amino acids, lipids, fatty acids, were found to contribute to the classification between patients and controls (as shown in Figure 5). Of these, 13 metabolites were shown to contribute to the differentiation between patients with benign SPNs and patients with malignant SPNs (*p* < 0.05 and AUC ≥ 0.7, as shown in the Supplementary Table).

*: Significant at *p* < 0.05 between malignant and benign SPNs.

### 2.3. Discussion

The strength of metabolomics is to assess dynamic metabolic responses in a comprehensive and high-throughput manner. Currently, GC/MS and LC/MS are the most frequently employed metabolomics techniques, with different and complementary capabilities. GC/MS has proven to be a powerful metabolomics tool, especially for the metabolites of low boiling points, such as amino acids, fatty acids and other organic acids, although it requires prior derivatization of most metabolites during the analysis [11]. In contrast, LC/MS, which does not require the time-consuming derivatization procedure and is suitable for analysis of the phosphorylated compounds, has become a promising analytical approach due to its rapid, sensitive, and wide range analytical capability. In previous studies, it was found that an integrated use of GC/MS and LC/MS methods could provide a complementary and significantly improved analytical strategy with excellent stability and reproducibility [18,19,20].

Our data showed that GC/MS and LC/MS measurements yielded distinct but inherently relevant metabolic profiles in plasma of patients with benign or malignant SPNs, which achieved a maximum coverage of the metabolome. The data obtained from either GC/MS or LC/MS exhibited a clear separation between cases and healthy controls. Besides, there was also a good separation between patients with benign SPNs and malignant SPNs, which might be very useful for their early discrimination in clinical practice. It is noted that most metabolites, derived either from GC/MS or LC/MS, were found to have the same change trend in patients with benign or malignant SPNs as compared to healthy controls. It thus implied that both benign and malignant SPNs share some common metabolic features during the course of disease insults. For example, the significantly decreased levels of amino acids (glycine, valine, isoleucine, glutamine, proline, threonine, and 5-hydroxytryptophan as well as L-arginine, deoxycholic acid glycine conjugate, *etc.*) were observed in both diseases, despite their pathological and histological features being so different. These amino acids have essential physiological properties and play pivotal roles in maintaining homeostasis. For instance, -hydroxytryptophan (5-HTP) synthesis is dependent on the rate of lung metabolism [21]. The decreased levels of 5-HTP may be reflective of impairment of lung function, which could be caused by the development of either benign or malignant SPNs. L-Arginine is deeply involved with the metabolism of nitric oxide (NO), a well-studied vasodilator and free radical involved in a variety of lung diseases [22,23]. The decreased levels of L-arginine were assumed to correspond to the increased NO levels, which have been confirmed in numerous earlier studies [24,25].

It is known that phospholipids are important constituents for maintaining cell structure and organelle integrity and also play a major role in energy and signaling pathways. Normally, phospholipids are protective and their concentrations could decline significantly during the course of various diseases [26]. Therefore it is not surprising to observe the decrease in phospholipids levels in plasma of patients with either benign or malignant SPNs, such as LPCs, LPEs, PCs, PEs, PSs. It is also well known that most of phospholipids are made up of fatty acids. Therefore, it is not surprising to see a remarkable increase in the levels of various fatty acids such as palmitic acid, oleic acid, linoleic acid, oxooctadecanoic acid in both benign and malignant SPNs.

With respect to other identified metabolites, inositol functions as the basis of a number of signaling and secondary messenger molecules which are involved in a number of biological processes. The decreased levels of inositol in both benign and malignant SPNs indicated that a common biological pathway could have been caused by dysfunction. In addition, bilirubin is one of the well-known by-products generated during heme degradation and is assumed to have antioxidant and anti-inflammatory roles in a variety of stress conditions [27]. Both bilirubin and biliverdin levels have been widely reported to increase considerably in various inflammatory diseases or cancers [28]. Taken together, the common metabolic disturbances we identified in either benign or malignant SPNs represent the self-defense mechanisms or anti-oxidative, anti-inflammatory pathways that are damaged or activated during the course of a variety of diseases.

Nevertheless, it should be noted that there were also 14 metabolites identified to contribute to the differentiation between benign and malignant SPNs in our study. These metabolites included N-succinyl-2,6-diaminopimelate, deoxycholic acid glycine conjugate, octanoylcarnitine, LPCs, LPE (20:2), PC (34:1), PSs, and cholesteryl acetate. The discovery of these metabolites indicated much more disturbed amino acid metabolism, lipid metabolism and cholesterol metabolism in malignant SPNs patients compared with patients with benign SPNs. Thus, metabolic profiles containing a group of these metabolites might be more effective in discriminating cancer patients and more informative to elucidate the progression of malignant SPNs.

## 3. Experimental Section

### 3.1. Clinical Populations

Seventeen male patients with malignant SPNs (age range 49–67) and 15 male patients with benign SPNs (age ranged between 33–79) and 20 male healthy controls (age range 29–50) were included in this study. Blood samples were collected around 9 am in the morning from both patients and healthy volunteers after overnight fasting. The detailed information of patients and healthy controls is summarized in Table 1. All patients had been diagnosed histologically either as malignant SPNs (lung squamous cell carcinoma at stage I) or benign SPNs at Shanghai Pulmonary Hospital, Tongji University School of Medicine, Shanghai, P.R. China. For histological analysis, tissue specimens were resected surgically and fixed in 10% neutral formalin, embedded in paraffin, cut into 5 m sections, and stained with hematoxylin and eosin (Figure 1).

All subjects gave their informed consent for inclusion before they participated in the study. The study was conducted in accordance with the Declaration of Helsinki, and the protocol was approved by the Ethics Committee of Shanghai Pulmonary Hospital. Patients provided blood samples preoperatively and none of them had undergone radiation or chemotherapy treatment or other medical treatment. Controls were healthy volunteers with no obvious illness who agreed to provide blood samples, health information and written informed consent. All of the participant’s demographics (age and smoking habits) and health status were documented.

### 3.2. Plasma sample Collection

Blood samples were collected into tubes containing EDTA as an anticoagulant. They were immediately placed on ice and transported to the laboratory. Samples were then centrifuged at 3,000 × g at 4 °C for 10 min. Plasma was separated, aliquoted and stored at -80°C for future analysis.

### 3.3. Chemicals

N-(9-Fluorenylmethoxycarbonyl)-glycine (FMOC-glycine) and N-methyl-N-trimethyl-silyl-trifluoroacetamide (MSTFA) were purchased from Sigma-Aldrich (St. Louis, MO, USA). Formic acid, pyridine and HPLC grade methanol and ethanol were purchased from Merck (Darmstadt, Germany). Deionised water was obtained from Millipore Milli-Q purification system (Bedford, MA, USA). All other reagents and solvents were of commercially available analytical grades.

### 3.4. Sample Pretreatment

A 350 µL ice-cold methanol (spiked with 10 µg/mL FMOC-glycine as internal standard, IS) was added to 50 µL plasma sample and vortexed for 2 min. The mixture was centrifuged twice (16,000 × g at 4 °C for 10 min), and then the supernatant was divided into two parts: one 100 µL for LC/MS analysis directly and another 100 µL for GC/MS analysis, which was first transferred to a glass vial and evaporated to dryness and then derivatized by 100 µL methoxyamine (50 µg/mL in pyridine, 37 °C × 2 h) and followed by 100 µL MSTFA (37 °C × 16 h). After centrifugation (6,000 × g at 4 °C for 1 min), 1.0 µL of the supernatant was injected into GC/MS.

### 3.5. GC/MS Analysis

Derivatized sample (1.0 µL) was injected splitlessly with an Agilent 7683 Series autosampler (Agilent Technologies, Santa Clara, CA, USA) into an Agilent 6890 GC system equipped with an Agilent 5973 Mass Selective Detector. Separation was performed on a fused-silica capillary column HP-5MSI (30 m × 0.25 mm i.d., 0.25 µm film thickness). The inlet temperature was set at 250 °C. Helium was used as the carrier gas with a constant flow rate 1 mL/min through the column. The GC oven temperature was maintained at 70 °C for 1 min, and then increased to 250 °C at a rate of 10 °C/min and further increased at 25 °C /min to 300 °C and hold for 6 min. The total running time was 27 min. The transfer line temperature was set at 280 °C. Detection was achieved using MS in electron impact mode (70 eV) and full scan monitoring (m/z 50 to 550). The temperature of the ion source was set at 230 °C, and the quadrupole was set at 150°C. The solvent delayed time was set as 4.1 min.

### 3.6. LC/MS Analysis

Supernatant from cold-methanol treated specimen was used directly for LC/MS analysis. LC/MS analysis was performed on an Agilent 1290 UHPLC system (Waldbronn, Germany) coupled to 6540 quadrupole-time of flight (Q-TOF) mass detector equipped with a dual jet stream electrospray ionization source and managed by a MassHunter workstation. The column used for the separation was an Agilent rapid resolution HT zorbax SB-C18 (2.1 × 50 mm, 1.8 µM). The oven temperature was maintained at 50 °C. The gradient elution involved a mobile phase consisting of (A) 0.1% formic acid in water and (B) 0.1% formic acid in methanol. The initial condition was set at 5% of B. The following solvent gradient was applied: from 5% B to 90% B within 5 min, hold for 2.5 min and then to 100% B within 2.5 min and hold for 4 min. Flow rate was set at 0.4 mL/min and 2 µL of samples was injected. The electrospray ionization-mass spectrometry (ESI-MS) was acquired in positive and negative ion mode, respectively. The ion spray voltage and nozzle voltage were set at 4,000 V and 1,000 V, respectively. The drying gas and sheath gas temperatures were maintained at 325 °C and 350 °C, respectively. The drying gas and sheath gas flow rates were 10 L/min and 12 L/min, respectively. The nebulizer nitrogen gas flow rate was set at 55 psi. For full scan mode analysis spectra were stored from *m/z* 101 to 1,400 in centroid mode.

### 3.7. Data Preprocessing

The raw data acquired from GC/MS and LC/MS were firstly analyzed by the MassHunter Find Compounds by Chromatogram Deconvolution and Find Compounds by Molecular Feature algorithms (Agilent), respectively, for detection of the compounds. The data were then exported to the Mass Profiler Professional software (Agilent) for peak alignment, followed by normalization with the intensity of FMOC-glycine and baselining to median across all samples. The molecular features that existed in at least 80% of the samples in either group were retained. Data acquired from both columns were combined after the above pretreatment for the following chemometrics analysis.

### 3.8. Chemometrics Data Analysis

The preprocessed GC/MS and LC/MS data were firstly tested using Mann-Whitney-Wilcoxon test with MultiExperiment View V4.6.1 software [29]. Only those with *p*-value < 0.05 were selected and exported to Soft Independent Modeling of Class Analogy (SIMCA-P 11.0, Umetrics AB, Umea, Sweden) for analysis and visualization by multivariate statistical methods. Prior to OPLS-DA, all data were adjusted for confounders (age and smoking habits). The normalized expression values were obtained by fitting a linear regression model on the metabolites expression with health status and confounders as predictors, and from the fitted model the variation attributed to the confounders were identified and removed from the expression values to obtain the normalized expression values. A 7-fold cross-validation was applied to OPLS-DA and the reliabilities of models were further validated by permutation tests. The receiver operating characteristic (ROC) curve was drawn and area under curve (AUC) for the selected features was calculated with Origin 8.0.

### 3.9. Marker Metabolites Selection and Identification

The variable importance in projection (VIP) values reflects the importance of terms in the OPLS-DA model both with respect to Y, *i.e.*, its correlation to all the responses, and with respect to X (the projection). Therefore, in this study only metabolites with VIP > 1 were selected for further evaluation.

To extract a list of robust metabolites that are not influenced by the other two factors (age and smoking habits), the *p*-values that test for association between metabolites expression values and the various factors individually were computed. A linear regression model with health status, age and smoking habits as predictors was performed and the *p*-values were obtained non-parametrically via bootstrap with 1,000 iterations. Metabolites that are significantly associated with any of the other two factors (*i.e*., *p* < 0.05) were removed.

The selected marker metabolites were further identified by comparison of tandem mass spectra (MS/MS) and retention time (RT) with those of reference standards, and those available in libraries including NIST 08, HMDB [30], METLIN [31] and LIPID MAPS [32].

## 4. Conclusions

In summary, we presented an integrated metabolomics approach by combining GC/MS and LC/MS for solitary pulmonary nodules plasma profiling. Our data showed that mass spectrometry-based metabolomics was able to provide a new approach to differentiate these two types of lung disease. Further refining and validation of these metabolites will be carried out in the future in a larger sample size of patients.

## Supplementary Files

Supplementary File 1 Supplementary (XLS, 24 KB)
